# Contemporary Use of Sodium Glucose Co-Transporter 2 Inhibitors in Hospitalized Heart Failure Patients: A “Real-World” Experience

**DOI:** 10.3390/jcm13123562

**Published:** 2024-06-18

**Authors:** Andrew Xanthopoulos, Nikolaos Katsiadas, Grigorios Giamouzis, Kleoniki Vangelakou, Dimitris Balaskas, Michail Papamichalis, Angeliki Bourazana, Nikolaos Chrysakis, Sotirios Kiokas, Christos Kourek, Alexandros Briasoulis, Niki Skopeliti, Konstantinos P. Makaritsis, John Parissis, Ioannis Stefanidis, Dimitrios Magouliotis, Thanos Athanasiou, Filippos Triposkiadis, John Skoularigis

**Affiliations:** 1 Department of Cardiology, University Hospital of Larissa, 41100 Larissa, Greece; grgiamouzis@gmail.com (G.G.); kleonikivag@hotmail.com (K.V.); balaskasdim@gmail.com (D.B.); mic_pap@yahoo.gr (M.P.); angi3bou@gmail.com (A.B.); nikoschrisakis8@yahoo.gr (N.C.); n.skopeliti@gmail.com (N.S.); ftriposk@med.uth.gr (F.T.); iskoular@gmail.com (J.S.); 2 Department of Cardiology, Konstantopouleio General Hospital of Athens, 14233 Athens, Greece; nikos5189@hotmail.com; 3 Department of Cardiology, General Hospital of Larissa, 41221 Larissa, Greece; sotkiokas@gmail.com; 4 Department of Clinical Therapeutic, Alexandra Hospital, National and Kapodistrian University of Athens, 11528 Athens, Greece; chris.kourek.92@gmail.com (C.K.); alexbriasoulis@gmail.com (A.B.); 5 Department of Medicine & Research Laboratory of Internal Medicine, Faculty of Medicine, University of Thessaly, 41334 Larissa, Greece; makaritsis@gmail.com; 6 National Expertise Center of Greece in Autoimmune Liver Diseases, General University Hospital of Larissa, 41110 Larissa, Greece; 7 Emergency Medicine Department, Attikon University Hospital, National and Kapodistrian University of Athens, 10679 Athens, Greece; jparissis@yahoo.com; 8 Department of Nephrology, Faculty of Medicine, University of Thessaly, 41334 Larissa, Greece; stefanid@med.uth.gr; 9 Department of Cardiothoracic Surgery, University Hospital of Larissa, 41110 Larissa, Greece; dimitrios.magouliotis.18@alumni.ucl.ac.uk (D.M.); t.athanasiou@imperial.ac.uk (T.A.)

**Keywords:** hospitalization, heart failure, acute, sodium glucose co-transporter 2 inhibitors, outcomes

## Abstract

**Background/Objectives**: The aim of this study was to examine the association between in-hospital initiation of sodium glucose co-transporter 2 inhibitors (SGLT2is) and outcomes in hospitalized heart failure (HHF) patients utilizing data from a Greek center. **Methods**: The present work was a single-center, retrospective, observational study of consecutive HF patients hospitalized in a tertiary center. The study endpoint was all-cause mortality or HF rehospitalization. Univariate and multivariate Cox proportional-hazard models were conducted to investigate the association between SGLT2i administration at discharge and the study endpoint. **Results**: Sample consisted of 171 patients, 55 of whom (32.2%) received SGLT2is at discharge. Overall, mean follow-up period was 6.1 months (SD = 4.8 months). Patients who received SGLT2is at discharge had a 43% lower probability of the study endpoint compared to those who did not receive SGLT2is at discharge (HR = 0.57; 95% CI: 0.36–0.91; *p* = 0.018). After adjusting for age, gender, smoking, hemoglobin (Hgb), use of SGLT2is at admission, use of Angiotensin-Converting Enzyme Inhibitors (ACEI-Is)/Angiotensin Receptor Blockers (ARBs) at discharge and Sacubitril/Valsartan at discharge, the aforementioned result remained significant (HR = 0.38; 95% CI: 0.19–0.73; *p* = 0.004). The 55 patients who received SGLT2is at discharge were propensity score matched with the 116 patients who did not receive SGLT2is at discharge. Receiving SGLT2is at discharge continued to be significantly associated with a lower probability of the study endpoint (HR= 0.43; 95% CI: 0.20–0.89; *p* = 0.024). **Conclusions**: Initiation of SGLT2is in HHF patients may be associated with better outcomes.

## 1. Introduction

Acute heart failure (HF) refers to the rapid or gradual onset of symptoms and/or signs of HF that results in either the patients’ urgent visit to the emergency department (ED) or an unplanned hospital admission [1,2]. Although it can manifest as a first clinical episode of HF (new onset), signs usually occur as an acutely decompensated chronic HF [2]. Acute HF continues to be the leading reason for hospitalization in people over 65 years of age and it is associated with high rates of mortality and rehospitalization for HF [3]. More specifically, in Europe and the USA over 1 million patients are admitted for HF each year, with a rehospitalization rate that reaches 50% at six months and a one-year mortality rate reaching 30% [4]. This results in an overload of health systems and an increase in medical costs [5]. Despite the development of new innovative treatments for the management of chronic HF, in-hospital treatment for acute HF is mainly symptomatic and involves the use of decongestive drugs with loop diuretics [1,2,3]. Therefore, there is an urgent need to find new treatments that will improve outcomes in these patients.

Sodium glucose co-transporter 2 inhibitors (SGLT2is) are a class of drugs that act on the proximal tubule and cause glycosuria and natriuresis [6]. Although initially used to treat patients with diabetes mellitus, SGLT2is have shown beneficial effects in the management of patients with HF [6]. These agents have been shown in large randomized trials to reduce both cardiovascular mortality and readmissions in patients with chronic HF with either reduced or preserved left ventricular ejection fraction (LVEF) [7,8,9,10]. As a result, they have become an integral part of the management of patients with chronic HF regardless of LVEF and they are an important weapon in the quiver of clinicians [2]. Although their value in chronic HF is indisputable, their role in patients with acute HF dysregulation is still under investigation. Recent studies have shown beneficial effects from the rapid initiation of SGLT2is and especially empagliflozin in patients with hospitalized HF (HHF) [11,12]. The aim of this study was to examine the association of in-hospital initiation of SGLT2is and outcomes in HHF patients utilizing “real world” data.

## 2. Materials and Methods

### 2.1. Study Population

**Figure 1:**
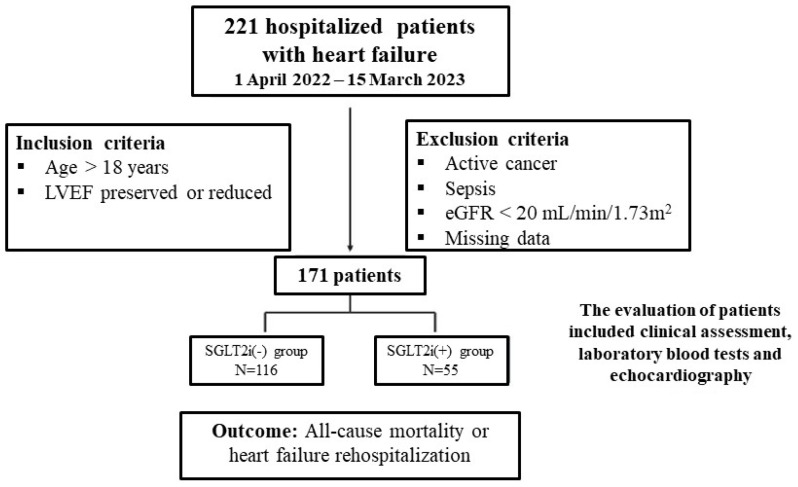
Study flowchart.

The present work was a single-center observational study of 221 single consecutive patients who were admitted in the Cardiology Department of the University Hospital of Larissa between 1 April 2022 and 15 March 2023. The data were obtained from the patients’ medical records. All patients had to be over 18 years of age with or without a known history of HF, regardless of LVEF. Patients with active cancer, sepsis, eGFR < 20 mL/min/1.73 m^2^ and missing data were excluded from the analysis. After the implementation of inclusion and exclusion criteria, the 171 patients that remained were divided into two cohorts. The first cohort consisted of 116 hospitalized HF patients who were not on SGLT2is at discharge and the second cohort consisted of 55 hospitalized HF patients who received SGLT2is at discharge (Figure 1).

This study was conducted in accordance with the Declaration of Helsinki and approved by the institutional review board of the University of Thessaly (protocol code: 53326; date of approval: 29 November 2023). Informed consent was waived due to the retrospective nature of the study.

### 2.2. Patient Assessment

The evaluation of patients at admission, during hospitalization and before discharge included a clinical assessment, laboratory blood tests and echocardiography. Levels of hematocrit (Ht), hemoglobin (Hgb) and Red Blood Cell Distribution Width (RDW) were measured with the use of a Unicel DxH 900 Hematology Analyzer (Beckman, USA) on samples obtained for standard of care evaluation, while urea, creatinine, serum glutamic oxaloacetic transaminase (SGOT), serum glutamic pyruvic transaminase (SGPT) and electrolytes were measured with the use of a Cobas 8000 (Roche, Germany). The echocardiography was reviewed by two independent echocardiographers with the use of a Vivid T8 v206 (General Electric Medical Systems, China). Standard echocardiographic measurements were obtained in accordance with the current guidelines of the European Association of Cardiovascular Imaging [13].

### 2.3. Outcomes

The outcome of the study was the combined endpoint of all-cause mortality or HF rehospitalization (whichever occurred first). Follow-up data were collected through outpatient clinic visits, telephone calls and death certificates.

### 2.4. Statistical Analysis

Quantitative variables were expressed as mean (Standard Deviation) or as median (interquartile range). Categorical variables were expressed as absolute and relative frequencies. For the comparison of proportions, chi-square and Fisher’s exact tests were used. Students’ *t*-tests and Mann–Whitney tests were used for the comparison of continuous variables between two groups. Kaplan–Meier survival curves for the study outcome were graphed over the follow-up period for the SGLT2i and non-SGLT2i at discharge groups. Univariate and multivariate Cox proportional-hazard models were conducted in order to find if receiving SGLT2is at discharge was significantly associated with all-cause mortality or HF rehospitalization. Moreover, the aforementioned Cox proportional-hazard model was also conducted in the subsample, where the 55 patients who received SGLT2is at discharge were propensity score matched, using a ratio 1:1, with the 116 patients who did not receive SGLT2is at discharge in order to ensure similar baseline characteristics. All reported *p* values are two-tailed. Statistical significance was set at *p* < 0.05 and analyses were conducted using SPSS statistical software (version 26.0).

## 3. Results

### 3.1. Patient Characteristics

Patients’ characteristics are presented in Table 1, in total sample and by administration of SGLT2is at discharge. Regarding the phenotypes of the HHF patients, 34 (20%) presented with acute pulmonary edema, 116 (68%) with acute decompensated HF, 14 (8%) with isolated right HF and 7 (4%) with cardiogenic shock. The percentage of males was significantly greater in the SGLT2i group vs. the no-SGLT2i group. Further, patients in the SGLT2i group were significantly younger and more frequently smokers. Hct and Hgb values were significantly higher in the SGLT2i group. Patients in the SGLT2i group were less frequently on ACE-Is/ARBs but were more often on Sac/Valsartan at discharge.

### 3.2. Study Outcomes

**Figure 2:**
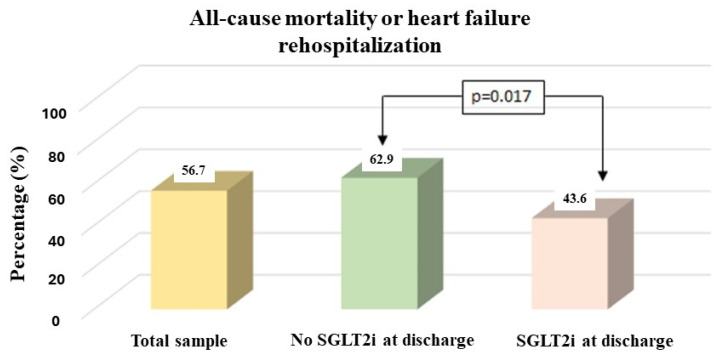
Percentages of all-cause mortality and heart failure rehospitalization, in total sample and by group.

More than half of the sample (56.7%) experienced HF rehospitalization or died. The aforementioned outcome in patients who received SGLT2is at discharge was significantly less frequent compared to those who did not receive SGLT2is at discharge (43.6% vs. 62.9%; *p* = 0.017; Figure 2).

**Figure 3:**
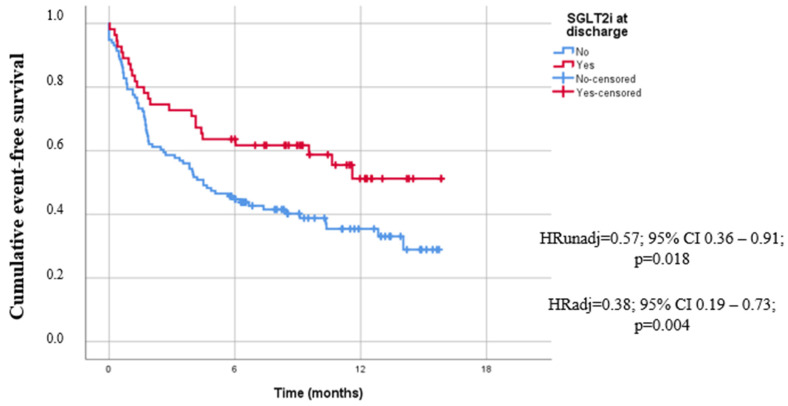
Kaplan–Meier curves for all-cause mortality or HF rehospitalization for patients receiving SGLT2is vs. no SGLT2is at discharge.

Patients who were on SGLT2is at discharge had a 43% lower probability of all-cause mortality or HF rehospitalization in comparison to those who were not on SGLT2is at discharge (Table 2, Figure 3).

After adjusting for age, gender, smoking, Hgb, administration of SGLT2is at admission, ACEI-Is/ARBs at discharge and Sacubitril/Valsartan at discharge, the aforementioned result remained significant, HR = 0.38; 95% CI: 0.19–0.73; *p* = 0.004.

Overall, mean follow-up period was 6.1 months (SD = 4.8 months). Mean overall event-free time was 8.20 months (SE = 0.52). For patients who received SGLT2is at discharge, mean event-free time was 10.03 months (SE = 0.90), and for those who did not receive SGLT2is at discharge, mean event-free time was significantly lower and equal to 7.29 months (SE = 0.61), *p* log-rank test = 0.018.

### 3.3. Subgroup Analysis

**Figure 4:**
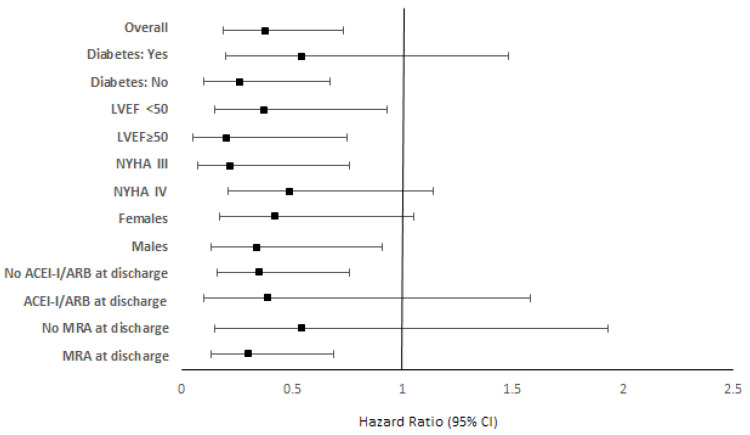
Adjusted hazard ratios (95% confidence intervals) for subgroup analyses. Abbreviations: LVEF, left ventricular ejection fraction; NYHA, New York Heart Association; ACE-Is, Angiotensin-Converting Enzyme Inhibitors; ARBs, Angiotensin Receptor Blockers; MRAs, Mineralocorticoid Receptor Antagonists.

When subgroup analysis was conducted, it was found that receiving SGLT2is at discharge was significantly associated with a significantly lower probability of all-cause mortality or HF rehospitalization in males, in patients without diabetes, those with NYHA III, those who did not receive ACEI-Is/ARBs at discharge and in those who received MRAs at discharge (Figure 4, Table 2). In addition, receiving SGLT2is at discharge was significantly associated with a significantly lower probability of all-cause mortality or HF rehospitalization regardless of patients’ LVEF. However, none of the interaction terms were found to be significant (*p* > 0.05), indicating that the effect of SGLT2i administration at discharge was similar within each subgroup.

### 3.4. Propensity Matching Analysis

The 55 patients who received SGLT2is at discharge were propensity score matched, using a ratio 1:1, with the 116 patients who did not receive SGLT2is at discharge. The propensity score matching was done using all baseline characteristics that differed between the two groups, i.e., age, gender, smoking, Hgb, use of SGLT2is at admission, ACEI-Is/ARBs at discharge and Sacubitril/Valsartan at discharge. Afterwards, 32 matching pairs of patients remained in the sample, whose characteristics are presented in Supplementary Table S1. In this sample, receiving SGLT2is at discharge continued to be significantly associated with a lower probability of all-cause mortality or HF rehospitalization, HR= 0.43; 95% CI: 0.20–0.89; *p* = 0.024 (Figure S1).

## 4. Discussion

In the present study, utilizing real world data from 171 consecutive HHF patients, we demonstrated that in-hospital initiation of SGLT2is was associated with a reduction in the composite endpoint of all-cause mortality or HF rehospitalization.

HF is a major health system problem which has been increasing in recent years, mainly due to the aging of the world population [14]. The natural course of HF syndrome is progressive, with periods of relative stabilization interspersed with periods of decompensation [1,4]. Acute HF refers to the rapid or gradual onset of HF signs and/or symptoms that prompt the patient to seek emergency medical attention [2]. This leads to either a visit to the ED or an unscheduled admission to the hospital [2]. Acute HF includes a wide range of clinical conditions with diverse etiologies and triggers [1,4]. It may involve either the first onset of HF or, more commonly, acute dysregulation of chronic stable HF [4,15]. Acute HF is a complex pathophysiological syndrome that includes several hemodynamic abnormalities associated with increased ventricular filling pressure and/or decreased cardiac output. Clinically, it manifests mainly with cardiac congestion, while hypoperfusion can also occur [4,15]. Patients presenting with acute HF have high rates of mortality and rehospitalization for HF decompensation [16]. In the present study, more than half of patients experienced HF rehospitalization or died during the follow-up.

Compared with chronic HF, there are fewer robust data to guide diagnosis, risk stratification and treatment in these patients [4]. Although new and innovative treatments have been developed for chronic HF in recent years, resulting in significant clinical benefits, in acute HF, treatment options are still limited [2].

Congestion is an important cause for cardiac decompensation, and therefore it is arguably considered a key therapeutic target in acute HF [4]. In fact, achieving complete decongestion at hospital discharge and maintaining it during the early period after discharge has been associated with better outcomes [17,18]. The main therapeutic option for the decongestion of these patients continues to be the use of diuretics and especially intravenous loop diuretics [17]. However, the management of these patients during hospitalization is still based on clinical expertise and experience [12,19].

One class of drugs that has shown great benefit in patients with chronic HF is SGLT2is [20]. SGLT2is act on the proximal convoluted tubule of the kidney and inhibit SGLT2 co-transporters, which causes a decrease in glucose reabsorption along the tubule, resulting in glycosuria [6,21]. Along with glycosuria, SGLT2 inhibition also causes natriuresis associated with negative salt and water balance [21]. Increased natriuresis and sodium delivery to the distal nephron is important for renal protection, as it normalizes the tubuloglomerular feedback mechanism [6]. Although initially used for their anti-diabetic properties, SGLT2is have resulted in great benefits in patients with chronic HF [2]. Large randomized trials on the use of SGLT2is in patients with chronic HF have showed a reduction in cardiovascular mortality and readmissions for HF decompensation [7,8,9,10,22]. These benefits were seen in both patients with reduced and preserved LVEF. The use of SGLT2is in patients with chronic HF is now an integral part and mainstay of treatment for all patients with chronic HF [2].

Although the role of SGLT2is in patients with chronic HF is well established through large randomized trials and meta-analyses, their role in patients with acute HF has not been fully established. There are some data showing a possible benefit of rapid initiation of SGLT2is in patients with HHF [11,12]. The first study that examined the effect of SGLT2is in patients with acute HF was SOLOIST-WHF [23]. This multi-center, double-blind, randomized study involved 1222 patients with type 2 DM who had recently been hospitalized for worsening HF and were randomized to receive sotagliflozin or a placebo. The aim was to examine the rate of cardiovascular death and rehospitalization for HF [23]. The group initiated on sotagliflozin before or soon after discharge had fewer cardiovascular deaths or rehospitalizations or emergency visits for HF over a 9-month follow-up period compared with the placebo group (245 vs. 355 events, HR 0.67, 95% CI 0.52–0.85). EMPA-RESPONSE-AHF was a randomized, placebo-controlled, double-blind pilot study in which 80 patients with acute HF with or without DM were randomized to receive empagliflozin 10 mg/day or a placebo for 30 days [24]. No significant differences were observed in the primary endpoints (change in visual analogue scale dyspnea score, diuretic response, change in NT-proBNP and length of stay). However, the empagliflozin group showed an increased in urinary output [difference 3449 (95% confidence interval 578–6321) mL; *p* <0.01] and a reduced combined endpoint of worsening HF, rehospitalization for HF or death at 60 days compared to the placebo group [4 (10%) vs. 13 (33%); *p* = 0.014] [24].

The most recent study that examined the effect of empagliflozin on decongestion in patients with acute HF was the EMPULSE trial [11]. In this randomized double-blind study, 530 patients who were hospitalized with acute HF with elevated NT-proBNP (≥1600 pg/mL) requiring at least 40 mg of iv furosemide per day and an eGFR ≥ 20 mL/min/m^2^ were randomized 1:1 to either empagliflozin 10 mg once daily or a placebo for 90 days [11]. The empagliflozin group resulted in an early, effective and sustained decongestion which was associated with clinical benefit at day 90. More specifically, using a win-ratio approach, empagliflozin significantly reduced the combined primary endpoint of death, the number of HF events, time to first HF event and change from baseline in KCCQ total symptom score at 90 days (clinical benefit of 53.9% vs. 39.7%, win ratio 1.36, 95% CI 1.09–1.68) [11]. In the present retrospective analysis, initiation of SGLT2is in HHF patients was associated with clinical benefits in these patients as it reduced deaths from any cause and HF readmissions.

Although SGLT2is in patients with chronic HF are part of first-line therapy, the mechanisms through which they exert their beneficial effects are still not fully clarified and even less is known about their actions in patients with HHF. For chronic HF, it has been hypothesized that the benefits of SGLT2is are mediated through pleiotropic actions involving metabolic, renal, cardiac and hemodynamic effects on the body. Regarding acute HF, the mechanism of action is more complicated [25,26]. The initiation of SGLT2is in patients with acute HF during their hospitalization or immediately after is suspected to lead to a rapid and sustained volume unloading and improvement of left ventricular filling pressure and diastolic function, which may contribute to the significant reduction in the risk of rehospitalization [27].

It is well documented that in patients with acute HF and volume overload, the first-line treatment is loop diuretics, aiming to produce natriuresis and a negative fluid balance [28]. Studies have shown that a higher urinary sodium concentration in patients with acute HF has been associated with better in-hospital and post-discharge outcomes [29,30]. However, the resulting volume depletion from loop diuretics can lead to activation of the renin–angiotensin–aldosterone system and the sympathetic nervous system, especially in tubular sites that cannot be acted upon by these diuretics, which can lead to the emergence of diuretic resistance [31,32]. This obstacle could be overcome by using a combination of diuretics that act at different sites, and SGLT2is could be a good choice since they act at a different site than loop diuretics to increase natriuresis [12]. Recent studies have shown that SGLT2i treatments in patients with acute HF increased urinary sodium excretion and increased diuresis, possibly due to the osmotic diuresis they cause [33,34]. Interestingly, SGLT2is are quite effective in removing fluid from the interstitial space rather than from the intravascular space, acting mainly as an anti-edematous agent without causing electrolyte disturbances [35]. Acute decompensated HF frequently causes kidney impairment and treatment may lead to acute kidney injury [36]. However, the use of SGLT2is in patients with acute HF appears to reduce the required doses of loop diuretics to decongest these patients, which may mitigate the risk of renal damage [37]. In a study by Thiele et al., it was shown that the use of empagliflozin in patients with acute decompensated HF did not affect hemodynamic parameters and instead significantly reduced markers of tubular injury, suggesting that SGLT2 inhibitor treatment may prevent acute kidney injury [38]. Although some studies have shown an initial decrease in eGFR in patients with acute HF receiving SGLT2is, this decrease appears to be transient as eGFR increased during follow-up [39]. The renoprotective effects of SGLT2 inhibitors have been attributed to kidney tubular–glomerular feedback with subsequent reduction of glomerular filtration pressure as the driving mechanism [38]. Another parameter possibly playing a role in the benefit of SGLT2is in acute HF is that they can lead to significantly lower NT-proBNP and BNP levels and a significantly higher incidence of hemoconcentration, all of which have been associated with a better prognosis [40,41].

Regarding the optimal timing for initiation of SGLT2is in patients with acute HF, it appears that an early initiation (within the first 5 days) is safe and effective as long as patients do not experience symptomatic hypotension or a need for inotropes or vasodilators within the previous six hours of their start [41]. Interestingly, in the present analysis approximately 1/3 of HHF received SGLT2is at discharge, which is in accordance with the current literature [42,43]. Lastly, since hospitalization is the result of progressive decompensation which occurs outside of the hospital, outpatient management is of utmost importance, including urgent ambulatory visits for the management of diuretics and optimal medical therapy (i.e., neurohormonal inhibitors and SGLT2is), to halt or reduce the worsening of the disease [44].

### Limitations

The major limitation associated with the present study is its observational nature; therefore, an inherent risk of confounding and bias cannot be excluded. However, different statistical methods (multiple adjustments and propensity matching) have been conducted to eliminate this risk. Furthermore, the studied sample was not large (171 consecutive patients) and the follow-up period was relatively short; however, each patient was presented once in this analysis. The number of patients with available NT-proBNP values at discharge was very small (*n* = 18, 10.5%), precluding any statistical analysis comparing cardiac biomarkers at admission versus at discharge in both subgroups. Lastly, the decision to initiate SGLT2is was up to the attending physician’s discretion, which may have led to a selection bias. Nevertheless, the aim of this study was to present “real world” data from a single center in Greece.

## 5. Conclusions

In the present “real world” analysis, the in-hospital initiation of SGLT2is in HHF patients was associated with improved prognosis. Further efforts are needed to overcome implementation barriers and improve use of SGLT2is among HHF patients.

## Figures and Tables

**Table 1:** Sample characteristics in total sample and by group.

	Total Sample (1 April 2022–15 March 2023) (*n* = 171; 100%)	SGLT2i (at Discharge)		
		No (*n* = 116; 67.8%)	Yes (*n* = 55; 32.2%)	
	*n* (%)	*n* (%)	*n* (%)	*p*
Gender				
Females	83 (48.5)	63 (54.3)	20 (36.4)	0.028 +
Males	88 (51.5)	53 (45.7)	35 (63.6)	
Age, mean (SD)	76 (11.8)	78.2 (11.4)	71.5 (11.4)	<0.001 ‡
SBP, mean (SD)	132.5 (28.9)	131.6 (27.9)	134.4 (30.9)	0.562 ‡
DBP, mean (SD)	77.4 (15.7)	77.2 (16.1)	77.7 (15.1)	0.859 ‡
Pulse Rate, mean (SD)	82.5 (20.4)	83 (20.9)	81.6 (19.7)	0.696 ‡
NYHA				
ΙΙΙ	59 (40.7)	38 (40.0)	21 (42.0)	0.816 +
ΙV	86 (59.3)	57 (60.0)	29 (58.0)	
LVEF, median (IQR)	35 (22.5–47.5)	35 (25–50)	35 (20–45)	0.180 ‡‡
HFrEF/HFmrEF/HFpEF				
HFrEF	103 (65.2)	63 (59.4)	40 (76.9)	0.094 +
HFmrEF	10 (6.3)	8 (7.5)	2 (3.8)	
HFpEF	45 (28.5)	35 (33)	10 (19.2)	
E/e’ (echocardiography)	14 (12–16)	14 (12–15)	14 (11.5–16)	0.764 ‡‡
NT-proBNP, median (IQR)	6490 (3410–10,500)	6520 (3185–10,350)	6385 (3880–10,800)	0.849 ‡‡
Cancer	9 (5.3)	5 (4.3)	4 (7.3)	0.471 ++
Hypertension	164 (95.9)	112 (96.6)	52 (94.5)	0.682 ++
Diabetes	67 (39.2)	46 (39.7)	21 (38.2)	0.854 +
COPD	12 (7)	10 (8.6)	2 (3.6)	0.342 ++
Dyslipidemia	116 (67.8)	80 (69)	36 (65.5)	0.646 +
Coronary disease	85 (49.7)	56 (48.3)	29 (52.7)	0.587 +
eGFR	60 (37–83)	56 (36–79.5)	64 (42–85)	0.138 ‡‡
Anemia	121 (70.8)	87 (75)	34 (61.8)	0.077 +
Atrial fibrillation	94 (55)	64 (55.2)	30 (54.5)	0.939 +
Obstructive Sleep Apnea	2 (1.2)	1 (0.9)	1 (1.8)	0.541 ++
Smoking				
Yes	16 (9.4)	7 (6.1)	9 (16.4)	0.032 +
No	130 (76.5)	88 (76.5)	42 (76.4)	
In the past	24 (14.1)	20 (17.4)	4 (7.3)	
Ht, mean (SD)	37.2 (6)	36.4 (5.6)	38.8 (6.5)	0.011 ‡
Hgb, mean (SD)	11.9 (2)	11.6 (1.9)	12.5 (2.2)	0.008 ‡
RDW, median (IQR)	16.2 (14.6–18.4)	16.5 (14.7–18.8)	15.5 (14.4–17.2)	0.075 ‡‡
Creatinine, median (IQR)	1.1 (0.9–1.6)	1.1 (0.9–1.6)	1.1 (0.9–1.5)	0.708 ‡‡
Urea, median (IQR)	51 (38.5–79)	55.5 (38.9–78.5)	47 (37–79.2)	0.442 ‡‡
Κ^+^, median (IQR)	4.5 (4.1–5)	4.5 (4.2–5)	4.4 (3.9–4.9)	0.160 ‡‡
Na^+^, median (IQR)	138 (134–140)	137.1 (133.1–140)	138.2 (136–140)	0.089 ‡‡
SGOT, median (IQR)	21 (16–31.4)	21.4 (16.4–32)	21 (15.2–29)	0.223 ‡‡
SGPT, median (IQR)	16 (11–24)	16 (10–23.5)	16 (12–25)	0.662 ‡‡
ACEI-Is/ARBs (at admission) *	58 (33.9)	43 (37.1)	15 (27.3)	0.206 +
B-Blocker (at admission) *	127 (74.3)	89 (76.7)	38 (69.1)	0.286 +
MRAs (at admission) *	57 (33.3)	42 (36.2)	15 (27.3)	0.247 +
Furosemide (at admission) *	104 (60.8)	76 (65.5)	28 (50.9)	0.068 +
Sacubitril/Valsartan (at admission) *	21 (12.3)	15 (12.9)	6 (10.9)	0.707 +
SGLT2i (at admission) *	23 (13.5)	0 (0)	23 (41.8)	<0.001 +
Inotropes/Vasopressors (during hospitalization)	20 (11.7)	13 (11.2)	7 (12.7)	0.776 +
ACE-Is/ARBs (at discharge)	58 (33.9)	47 (40.5)	11 (20)	0.008 +
B-Blocker (at discharge)	151 (88.3)	100 (86.2)	51 (92.7)	0.215 +
MRAs (at discharge)	113 (66.1)	72 (62.1)	41 (74.5)	0.107 +
Furosemide (at discharge)	146 (85.4)	99 (85.3)	47 (85.5)	0.985 +
Sacubitril/Valsartan (at discharge)	43 (25.1)	18 (15.5)	25 (45.5)	<0.001 +
ICD/CRT	33 (19.2)	22 (18.9)	11 (20)	0.865 +
All-cause death or HF rehospitalization	97 (56.7)	73 (62.9)	24 (43.6)	0.017 +

+ Pearson’s chi-square test; ++ Fisher’s exact test; ‡ Student’s *t*-test; ‡‡ Mann–Whitney test. * Patient’s previous medical treatment when arrived at the hospital. Abbreviations: ACE-Is, Angiotensin Converting Enzyme Inhibitors; ARBs, Angiotensin Receptor Blockers; COPD, Chronic Obstructive Pulmonary Disease; CRT, Cardiac Resynchronization Therapy; DBP, Diastolic Blood Pressure; eGFR, estimated Glomerular Filtration Rate; HFmrEF, Heart Failure with mildly reduced Ejection Fraction; HFpEF, Heart Failure with Preserved Ejection Fraction; HFrEF, Heart Failure with Reduced Ejection Fraction; Ht, hematocrit; Hgb, hemoglobin; ICD, Implantable Cardioverter Defibrillator; LVEF, left ventricular ejection fraction; NYHA, New York Heart Association; MRAs, Mineralocorticoid Receptor Antagonists; NT-proBNP, N-terminal pro B-type natriuretic peptide; RDW, Red Blood Cell Distribution Width; SBP, Systolic Blood Pressure; SGLT2is, sodium glucose co-transporter 2 inhibitors; SGOT, serum glutamic oxaloacetic transaminase; SGPT, serum glutamic pyruvic transaminase.

**Table 2:** Cox regression results for patients’ all-cause mortality and HF rehospitalization as dependent variable and administration of SGLT2is at discharge as independent variable.

	HR (95% CI) ^1^	*p*	HR (95% CI) ^2^	*p*	*p* Interaction Term ^3^
Total sample	0.57 (0.36–0.91)	0.018	0.38 (0.19–0.73)	0.004	-
Diabetes: Yes	0.69 (0.33–1.47)	0.341	0.54 (0.20–1.48)	0.229	0.612
Diabetes: No	0.51 (0.28–0.91)	0.023	0.26 (0.10–0.67)	0.005	
LVEF < 50	0.56 (0.31–0.99)	0.049	0.37 (0.15–0.93)	0.035	0.768
LVEF ≥ 50	0.47 (0.16–1.42)	0.182	0.20 (0.05–0.75)	0.017	
NYHA: ΙΙΙ	0.41 (0.17–1.02)	0.054	0.22 (0.07–0.76)	0.017	0.317
NYHA: ΙV	0.73 (0.40–1.31)	0.285	0.49 (0.21–1.14)	0.098	
Gender: Females	0.44 (0.20–0.99)	0.046	0.42 (0.17–1.05)	0.065	0.485
Gender: Males	0.64 (0.36–1.15)	0.139	0.34 (0.13–0.91)	0.031	
ACEI-Is/ARBs (discharge): No	0.53 (0.31–0.89)	0.017	0.35 (0.16–0.76)	0.008	0.949
ACEI-Is/ARBs (discharge): Yes	0.53 (0.18–1.52)	0.235	0.39 (0.10–1.58)	0.188	
MRAs (discharge): No	0.89 (0.40–2.00)	0.777	0.54 (0.15–1.93)	0.344	0.179
MRAs (discharge): Yes	0.47 (0.26–0.82)	0.008	0.30 (0.13–0.69)	0.005	

^1^ Unadjusted hazard ratio (95% confidence interval) for use of SGLT2is at discharge. ^2^ Hazard ratio (95% confidence interval) for receiving SGLT2is at discharge adjusted for age, gender (except for when analysis was conducted separately in males and females), smoking, Hgb, receiving SGLT2is at admission, ACEI-Is/ARBs at discharge (except for when analysis was conducted separately in these groups) and Sacubitril/Valsartan at discharge. ^3^ Interaction term for testing if the effect of receiving SGLT2is at discharge was different within each subgroups’ levels. Abbreviations: As in Table 1.

## Data Availability

The original contributions presented in the study are included in the Article/Supplementary Materials, further inquiries can be directed to the corresponding author.

## Supplementary Materials

The following supporting information can be downloaded at: https://www.mdpi.com/article/10.3390/jcm13123562/s1, Figure S1: Kaplan–Meier curves for patients receiving vs. not receiving SGLT2is at discharge in the propensity score matched sample; Table S1: Sample characteristics after propensity matching, by administration of SGLT2is at discharge.

## References

[1] Arrigo M., Jessup M., Mullens W., Reza N., Shah A.M., Sliwa K., Mebazaa A. (2020). Acute heart failure. Nat. Rev. Dis. Primers.

[2] McDonagh T.A., Metra M., Adamo M., Gardner R.S., Baumbach A., Bohm M., Burri H., Butler J., Celutkiene J., Chioncel O. (2021). 2021 ESC Guidelines for the diagnosis and treatment of acute and chronic heart failure. Eur. Heart J..

[3] Chioncel O., Mebazaa A., Harjola V.P., Coats A.J., Piepoli M.F., Crespo-Leiro M.G., Laroche C., Seferovic P.M., Anker S.D., Ferrari R. (2017). Clinical phenotypes and outcome of patients hospitalized for acute heart failure: The ESC Heart Failure Long-Term Registry. Eur. J. Heart Fail..

[4] Njoroge J.N., Teerlink J.R. (2021). Pathophysiology and Therapeutic Approaches to Acute Decompensated Heart Failure. Circ. Res..

[5] Kwok C.S., Abramov D., Parwani P., Ghosh R.K., Kittleson M., Ahmad F.Z., Al Ayoubi F., Van Spall H.G.C., Mamas M.A. (2021). Cost of inpatient heart failure care and 30-day readmissions in the United States. Int. J. Cardiol..

[6] Fonseca-Correa J.I., Correa-Rotter R. (2021). Sodium-Glucose Cotransporter 2 Inhibitors Mechanisms of Action: A Review. Front. Med..

[7] McMurray J.J.V., Solomon S.D., Inzucchi S.E., Kober L., Kosiborod M.N., Martinez F.A., Ponikowski P., Sabatine M.S., Anand I.S., Belohlavek J. (2019). Dapagliflozin in Patients with Heart Failure and Reduced Ejection Fraction. N. Engl. J. Med..

[8] Anker S.D., Butler J., Filippatos G., Ferreira J.P., Bocchi E., Bohm M., Brunner-La Rocca H.P., Choi D.J., Chopra V., Chuquiure-Valenzuela E. (2021). Empagliflozin in Heart Failure with a Preserved Ejection Fraction. N. Engl. J. Med..

[9] Packer M., Anker S.D., Butler J., Filippatos G., Pocock S.J., Carson P., Januzzi J., Verma S., Tsutsui H., Brueckmann M. (2020). Cardiovascular and Renal Outcomes with Empagliflozin in Heart Failure. N. Engl. J. Med..

[10] Solomon S.D., McMurray J.J.V., Claggett B., de Boer R.A., DeMets D., Hernandez A.F., Inzucchi S.E., Kosiborod M.N., Lam C.S.P., Martinez F. (2022). Dapagliflozin in Heart Failure with Mildly Reduced or Preserved Ejection Fraction. N. Engl. J. Med..

[11] Voors A.A., Angermann C.E., Teerlink J.R., Collins S.P., Kosiborod M., Biegus J., Ferreira J.P., Nassif M.E., Psotka M.A., Tromp J. (2022). The SGLT2 inhibitor empagliflozin in patients hospitalized for acute heart failure: A multinational randomized trial. Nat. Med..

[12] Carvalho P.E.P., Veiga T.M.A., Simoes E.S.A.C., Gewehr D.M., Dagostin C.S., Fernandes A., Nasi G., Cardoso R. (2023). Cardiovascular and renal effects of SGLT2 inhibitor initiation in acute heart failure: A meta-analysis of randomized controlled trials. Clin. Res. Cardiol..

[13] Lang R.M., Badano L.P., Mor-Avi V., Afilalo J., Armstrong A., Ernande L., Flachskampf F.A., Foster E., Goldstein S.A., Kuznetsova T. (2015). Recommendations for cardiac chamber quantification by echocardiography in adults: An update from the American Society of Echocardiography and the European Association of Cardiovascular Imaging. Eur. Heart J. Cardiovasc. Imaging.

[14] Savarese G., Lund L.H. (2017). Global Public Health Burden of Heart Failure. Card. Fail. Rev..

[15] Sinnenberg L., Givertz M.M. (2020). Acute heart failure. Trends Cardiovasc. Med..

[16] Kitakata H., Kohno T., Kohsaka S., Shiraishi Y., Parizo J.T., Niimi N., Goda A., Nishihata Y., Heidenreich P.A., Yoshikawa T. (2020). Prognostic Implications of Early and Midrange Readmissions After Acute Heart Failure Hospitalizations: A Report from a Japanese Multicenter Registry. J. Am. Heart Assoc..

[17] Hodson D.Z., Griffin M., Mahoney D., Raghavendra P., Ahmad T., Turner J., Wilson F.P., Tang W.H.W., Rao V.S., Collins S.P. (2019). Natriuretic Response Is Highly Variable and Associated With 6-Month Survival: Insights from the ROSE-AHF Trial. JACC Heart Fail..

[18] Dimos A., Xanthopoulos A., Giamouzis G., Kitai T., Economou D., Skoularigis J., Triposkiadis F. (2023). The “vulnerable” post hospital discharge period in acutely decompensated chronic vs. De-Novo heart failure: Outcome prediction using the Larissa Heart Failure Risk Score. Hellenic J. Cardiol..

[19] Lopez-Vilella R., Jover Pastor P., Donoso Trenado V., Sanchez-Lazaro I., Martinez Dolz L., Almenar Bonet L. (2023). Clinical phenotypes according to diuretic combination in acute heart failure. Hellenic J. Cardiol..

[20] Tromp J., Ouwerkerk W., van Veldhuisen D.J., Hillege H.L., Richards A.M., van der Meer P., Anand I.S., Lam C.S.P., Voors A.A. (2022). A Systematic Review and Network Meta-Analysis of Pharmacological Treatment of Heart Failure with Reduced Ejection Fraction. JACC Heart Fail..

[21] Zelniker T.A., Braunwald E. (2020). Mechanisms of Cardiorenal Effects of Sodium-Glucose Cotransporter 2 Inhibitors: JACC State-of-the-Art Review. J. Am. Coll. Cardiol..

[22] Zannad F., Ferreira J.P., Pocock S.J., Anker S.D., Butler J., Filippatos G., Brueckmann M., Ofstad A.P., Pfarr E., Jamal W. (2020). SGLT2 inhibitors in patients with heart failure with reduced ejection fraction: A meta-analysis of the EMPEROR-Reduced and DAPA-HF trials. Lancet.

[23] Bhatt D.L., Szarek M., Steg P.G., Cannon C.P., Leiter L.A., McGuire D.K., Lewis J.B., Riddle M.C., Voors A.A., Metra M. (2021). Sotagliflozin in Patients with Diabetes and Recent Worsening Heart Failure. N. Engl. J. Med..

[24] Damman K., Beusekamp J.C., Boorsma E.M., Swart H.P., Smilde T.D.J., Elvan A., van Eck J.W.M., Heerspink H.J.L., Voors A.A. (2020). Randomized, double-blind, placebo-controlled, multicentre pilot study on the effects of empagliflozin on clinical outcomes in patients with acute decompensated heart failure (EMPA-RESPONSE-AHF). Eur. J. Heart Fail..

[25] Patel D.K., Strong J. (2019). The Pleiotropic Effects of Sodium-Glucose Cotransporter-2 Inhibitors: Beyond the Glycemic Benefit. Diabetes Ther..

[26] Seferovic P.M., Fragasso G., Petrie M., Mullens W., Ferrari R., Thum T., Bauersachs J., Anker S.D., Ray R., Cavusoglu Y. (2020). Sodium-glucose co-transporter 2 inhibitors in heart failure: Beyond glycaemic control. A position paper of the Heart Failure Association of the European Society of Cardiology. Eur. J. Heart Fail..

[27] Salah H.M., Al’Aref S.J., Khan M.S., Al-Hawwas M., Vallurupalli S., Mehta J.L., Mounsey J.P., Greene S.J., McGuire D.K., Lopes R.D. (2022). Efficacy and safety of sodium-glucose cotransporter 2 inhibitors initiation in patients with acute heart failure, with and without type 2 diabetes: A systematic review and meta-analysis. Cardiovasc. Diabetol..

[28] Mullens W., Damman K., Harjola V.P., Mebazaa A., Brunner-La Rocca H.P., Martens P., Testani J.M., Tang W.H.W., Orso F., Rossignol P. (2019). The use of diuretics in heart failure with congestion—A position statement from the Heart Failure Association of the European Society of Cardiology. Eur. J. Heart Fail..

[29] Honda S., Nagai T., Nishimura K., Nakai M., Honda Y., Nakano H., Iwakami N., Sugano Y., Asaumi Y., Aiba T. (2018). Long-term prognostic significance of urinary sodium concentration in patients with acute heart failure. Int. J. Cardiol..

[30] Tersalvi G., Dauw J., Gasperetti A., Winterton D., Cioffi G.M., Scopigni F., Pedrazzini G., Mullens W. (2021). The value of urinary sodium assessment in acute heart failure. Eur. Heart J. Acute Cardiovasc. Care.

[31] Mullens W., Verbrugge F.H., Nijst P., Tang W.H.W. (2017). Renal sodium avidity in heart failure: From pathophysiology to treatment strategies. Eur. Heart J..

[32] Gupta R., Testani J., Collins S. (2019). Diuretic Resistance in Heart Failure. Curr. Heart Fail. Rep..

[33] Schulze P.C., Bogoviku J., Westphal J., Aftanski P., Haertel F., Grund S., von Haehling S., Schumacher U., Mobius-Winkler S., Busch M. (2022). Effects of Early Empagliflozin Initiation on Diuresis and Kidney Function in Patients with Acute Decompensated Heart Failure (EMPAG-HF). Circulation.

[34] Tamaki S., Yamada T., Watanabe T., Morita T., Furukawa Y., Kawasaki M., Kikuchi A., Kawai T., Seo M., Abe M. (2021). Effect of Empagliflozin as an Add-On Therapy on Decongestion and Renal Function in Patients with Diabetes Hospitalized for Acute Decompensated Heart Failure: A Prospective Randomized Controlled Study. Circ. Heart Fail..

[35] Hallow K.M., Helmlinger G., Greasley P.J., McMurray J.J.V., Boulton D.W. (2018). Why do SGLT2 inhibitors reduce heart failure hospitalization? A differential volume regulation hypothesis. Diabetes Obes. Metab..

[36] Filippatos G., Farmakis D., Parissis J. (2014). Renal dysfunction and heart failure: Things are seldom what they seem. Eur. Heart J..

[37] Shirakabe A., Matsushita M., Kiuchi K., Okazaki H., Inami T., Takayasu T., Asano M., Nomura A., Kobayashi N., Okajima F. (2020). Empagliflozin Administration Can Decrease the Dose of Loop Diuretics and Prevent the Exacerbation of Renal Tubular Injury in Patients with Compensated Heart Failure Complicated by Diabetes. Circ. Rep..

[38] Thiele K., Rau M., Hartmann N.K., Moller M., Mollmann J., Jankowski J., Keszei A.P., Bohm M., Floege J., Marx N. (2022). Empagliflozin reduces markers of acute kidney injury in patients with acute decompensated heart failure. ESC Heart Fail..

[39] Ul Amin N., Sabir F., Amin T., Sarfraz Z., Sarfraz A., Robles-Velasco K., Cherrez-Ojeda I. (2022). SGLT2 Inhibitors in Acute Heart Failure: A Meta-Analysis of Randomized Controlled Trials. Healthcare.

[40] Maisel A., Mueller C., Adams K., Anker S.D., Aspromonte N., Cleland J.G., Cohen-Solal A., Dahlstrom U., DeMaria A., Di Somma S. (2008). State of the art: Using natriuretic peptide levels in clinical practice. Eur. J. Heart Fail..

[41] Monzo L., Ferrari I., Cicogna F., Tota C., Cice G., Girerd N., Calo L. (2023). Sodium-glucose co-transporter 2 inhibitors in heart failure: An updated evidence-based practical guidance for clinicians. Eur. Heart J. Suppl..

[42] Pierce J.B., Vaduganathan M., Fonarow G.C., Ikeaba U., Chiswell K., Butler J., DeVore A.D., Heidenreich P.A., Huang J.C., Kittleson M.M. (2023). Contemporary Use of Sodium-Glucose Cotransporter-2 Inhibitor Therapy Among Patients Hospitalized for Heart Failure with Reduced Ejection Fraction in the US: The Get with The Guidelines-Heart Failure Registry. JAMA Cardiol..

[43] Parissis J., Georgiou C., Bistola V., Karavidas A., Vassilikos V.P., Kanakakis J., Davlouros P., Tziakas D.N., Alexanian I.P., Kochiadakis G. (2023). Rationale and Design of Heart Failure Prevalence and Evolution of Heart Failure in Diabetes Mellitus Type II Patients at High Risk (HF-LanDMark Study). J. Clin. Med..

[44] D’Amato A., Prosperi S., Severino P., Myftari V., Labbro Francia A., Cestiè C., Pierucci N., Marek-Iannucci S., Mariani M.V., Germanò R. (2024). Current Approaches to Worsening Heart Failure: Pathophysiological and Molecular Insights. Int. J. Mol. Sci..

