# Supplementary material for: Contemporary Use of Sodium Glucose Co-Transporter 2 Inhibitors in Hospitalized Heart Failure Patients: A “Real-World” Experience
Source: J Clin Med. 2024 Jun 18;13(12):3562. doi: 10.3390/jcm13123562 (PMC11204975; doi:10.3390/jcm13123562)
Supplement: Supplementary file 1 [file jcm-13-03562-s001.zip › jcm-3028021-supplementary.pdf]

Supplementary material

Figure S1. Kaplan-Meier curves for patients receiving vs not receiving SGLT2i at discharge in the propensity score matched sample

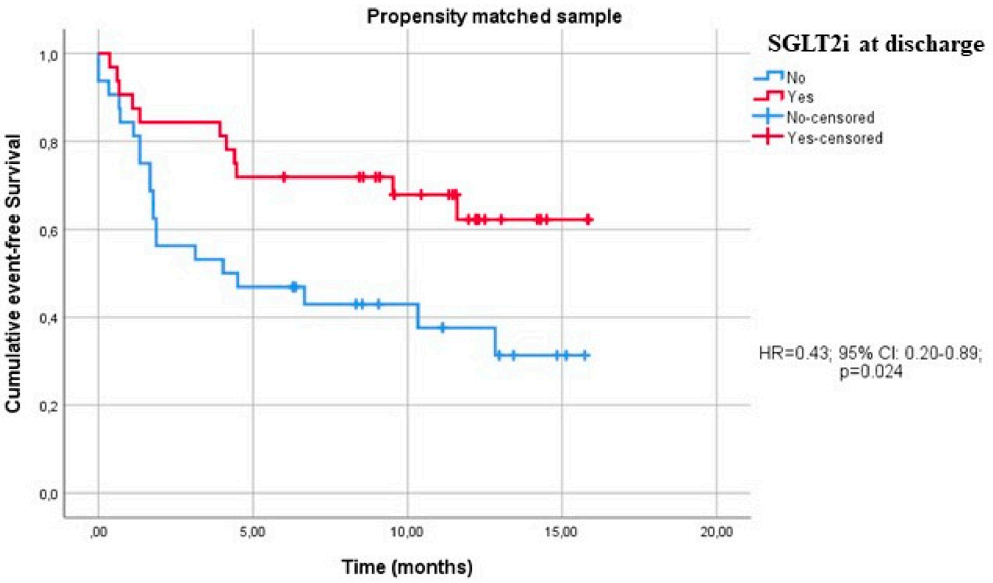

Table S1. Sample characteristics after propensity matching, by administration of SGLT2i at discharge

	Post-propensity score matching		
		SGLT2i (at discharge)	
	Total sample (n=64; 100%) n (%)	No (n=32; 67.8%) n (%)	Yes (n=32; 32.2%) n (%)
Propensity score matched characteristics			
Gender			
Females	31 (48.4)	15 (46.9)	16 (50)
Males	33 (51.6)	17 (53.1)	16 (50)
Age, mean (SD)	73.3 (11.1)	73.6 (10.8)	73 (11.5)
Smoking			
Yes	12 (18.8)	5 (15.6)	7 (21.9)
No	50 (78.1)	26 (81.3)	24 (75)
History	2 (3.1)	1 (3.1)	1 (3.1)
Hgb, mean (SD)	12.1 (1.9)	12.1 (1.7)	12.2 (2)
SGLT2i (at admission)	0 (0)	0 (0)	0 (0)
ACE-I/ARB (at discharge)	18 (28.1)	10 (31.3)	8 (25)
Sacubitril/Valsartan (at discharge)	33 (51.6)	17 (53.1)	16 (50)
Other baseline characteristics			
SBP, mean (SD)	129.6 (25.4)	126.6 (23.1)	132.5 (27.6)
DBP, mean (SD)	77 (13.8)	77.4 (15.2)	76.7 (12.6)
Pulse Rate, mean (SD)	83.7 (23.1)	82.6 (24.4)	84.9 (22)
NYHA			
III	30 (50.8)	15 (53.6)	15 (48.4)
IV	29 (49.2)	13 (46.4)	16 (51.6)
LVEF, median (IQR)	35 (20 — 45)	30 (20 — 40)	35 (30 — 50)
HFrEF/ HFmrEF/ HPpEF			
HFrEF	45 (73.8)	24 (80)	21 (67.7)
HFmrEF	2 (3.3)	1 (3.3)	1 (3.2)
HPpEF	14 (23)	5 (16.7)	9 (29)
NT-proBNP, median (IQR)	6240 (3180 — 10550)	5830 (3100 — 7530)	6620 (3700 — 10800)
Cancer	3 (4.7)	1 (3.1)	2 (6.3)
Hypertension	60 (93.8)	31 (96.9)	29 (90.6)
Diabetes	26 (40.6)	14 (43.8)	12 (37.5)
COPD	1 (1.6)	1 (3.1)	0 (0)
Dyslipidemia	37 (57.8)	19 (59.4)	18 (56.3)
Coronary Artery Disease	30 (46.9)	16 (50)	14 (43.8)
eGFR, median (IQR)	68 (47 — 85)	63.5 (38.5 — 79.5)	72.5 (58 — 86)
Anemia	45 (70.3)	24 (75)	21 (65.6)
Atrial fibrillation	36 (56.3)	18 (56.3)	18 (56.3)
Obstructive Sleep Apnea	1 (1.6)	0 (0)	1 (3.1)
Ht, mean (SD)	38 (5.5)	37.9 (5)	38.1 (5.9)
RDW, median (IQR)	16 (14.6 — 18.5)	17.2 (14.6 — 19.3)	15.5 (14.6 — 16.7)
Creatinine, median (IQR)	1.1 (0.8 — 1.3)	1.1 (0.9 — 1.5)	1 (0.8 — 1.2)
Urea, median (IQR)	44.8 (35 — 65.8)	45.3 (37 — 68.6)	44.6 (32.5 — 58.7)
K^+, median (IQR)	4.6 (4.2 — 5.1)	4.7 (4.4 — 5.4)	4.4 (3.9 — 4.9)

Na^+, median (IQR)	138 (134.7 — 140)	135.3 (132 — 140)	139 (137.6 — 140)
SGOT, median (IQR)	19.7 (15.7 — 30)	19.2 (16.4 — 31.5)	20.4 (14 — 28)
SGPT, median (IQR)	14.9 (9.5 — 22.7)	12 (8.5 — 20.3)	15.1 (12 — 29)
ACEI-I/ARB (at admission)	20 (31.3)	9 (28.1)	11 (34.4)
B-Blocker (at admission)	47 (73.4)	27 (84.4)	20 (62.5)
MRA (at admission)	21 (32.8)	16 (50)	5 (15.6)
Furosemide (at admission)	37 (57.8)	24 (75)	13 (40.6)
Sacubitril/Valsartan (at admission)	13 (20.3)	12 (37.5)	1 (3.1)
B-Blocker (at discharge)	57 (89.1)	29 (90.6)	28 (87.5)
MRA (at discharge)	48 (75)	23 (71.9)	25 (78.1)
Furosemide (at discharge)	53 (82.8)	26 (81.3)	27 (84.4)
Outcome			
All-cause mortality or HF rehospitalization	31 (48.4)	20 (62.5)	11 (34.4)

+Pearson's chi-square test; ++Fisher's exact test; ‡Student's t-test; ‡‡Mann-Whitney test

Abbreviations: ACE-I, Angiotensin Converting Enzyme Inhibitors; ARB, Angiotensin Receptor Blockers; COPD, Chronic Obstructive Pulmonary Disease; DBP, Diastolic Blood Pressure; eGFR, estimated Glomerular Filtration Rate; HFmrEF, Heart Failure with mildly reduced Ejection Fraction; HFpEF, Heart Failure with Preserved Ejection Fraction; HFrEF, Heart Failure with Reduced Ejection Fraction; Ht, Hematocrit; Hgb, Hemoglobin; NYHA, LVEF, Left Ventricular Ejection Fraction; New York Heart Association; MRA, Mineralocorticoid Receptor Antagonists; NT-proBNP, SBP, N-terminal pro B-type natriuretic peptide; RDW, Red Blood Cell Distribution Width; Systolic Blood Pressure; SGLT2i, Sodium Glucose Co-transporter 2 inhibitors; SGOT, Serum Glutamic Oxaloacetic Transaminase; SGPT, Serum Glutamic Pyruvic Transaminase
